# Diblock/Triblock Composition Controls Pore Structure and Mechanical Response in Hierarchically Ordered Hydrogels

**DOI:** 10.1021/acsapm.6c02944

**Published:** 2026-09-07

**Authors:** Elisabeth C. Lloyd, Sujata Dhakal, Sathika B. Arachchige, Mykyta Dementyev, Shubhra Goel, Svetlana Morozova, Robert J. Hickey

**Affiliations:** † Materials Science and Engineering, 311285 The Pennsylvania State University, University Park, Pennsylvania 16802, United States; ‡ Macromolecular Science and Engineering, 24575 Case Western Reserve University, Cleveland, Ohio 44106, United States; § Department of Chemistry, The Pennsylvania State University, University Park, Pennsylvania 16802, United States; ∥ Materials Research Institute, The Pennsylvania State University, University Park, Pennsylvania 16802, United States

**Keywords:** hydrogel, self-assembly, block copolymers, physical crosslinks, in situ deformation

## Abstract

Hydrogels are ubiquitous in a range of applications, including drug delivery, tissue engineering, and personal care products. The prevalence is a direct result of fundamental advances in the physics and chemistry of swollen networks that enable tailorable properties to enhance and promote favorable interactions with living tissues. An inherent challenge in hydrogel materials is controlling physicochemical properties such as macroscopic mechanical responses by simultaneously tuning molecular functionality and hierarchical structure. Here, we demonstrate control of macropore size and bulk mechanical properties in physically crosslinked, porous, hierarchically ordered hydrogels by tuning the diblock/triblock ratio. Specifically, a chemically identical diblock copolymer was blended with the corresponding triblock copolymer, leading to hydrogels with larger macroscopic pores that are softer, less tough, and fracture at lower extensions. In situ confocal measurements clearly demonstrate that pore deformation and alignment in the stretch direction under strain occurs at a larger degree for hydrogels prepared with lower triblock content, highlighting the relationship between pore morphology and mechanical response. The changes in mechanical properties and deformation response are attributed to the simultaneous effects of reducing the number of physical crosslinks and the formation of larger pores. The bottom-up self-assembly of porous, hierarchically ordered hydrogels demonstrating structure-dependent mechanical properties, as opposed to chemical modifications, adds an additional tuning handle to tailor material properties for desired applications.

## Graphical abstract

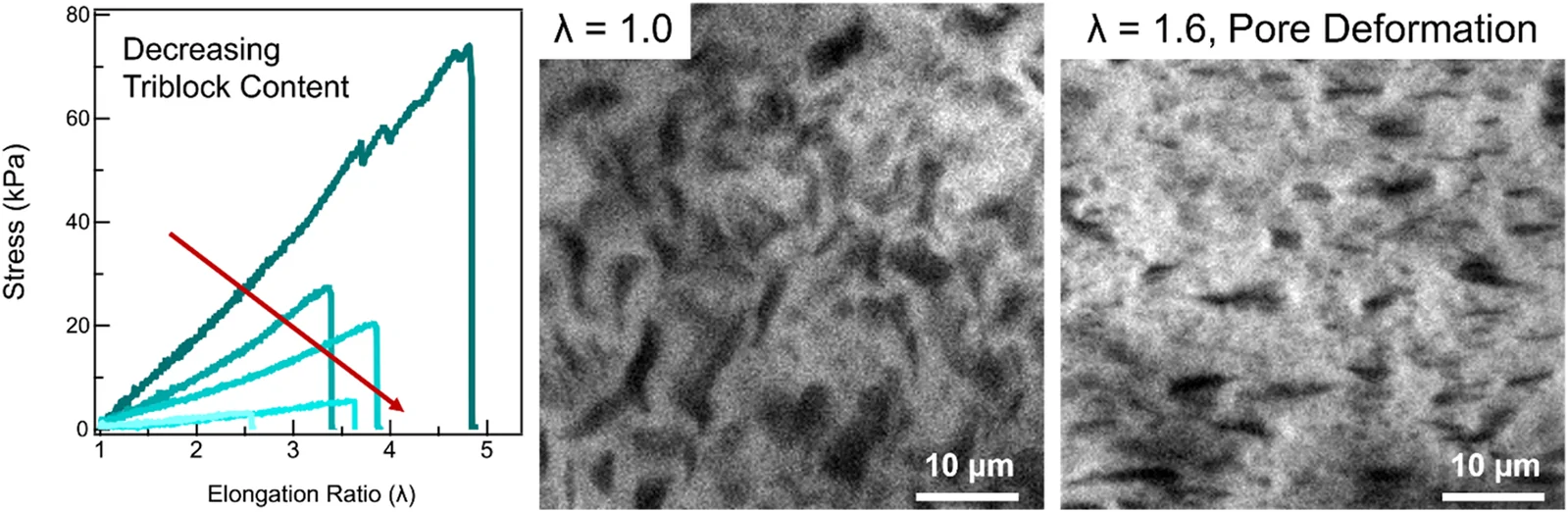

## Introduction

Hydrogels are critical materials for a wide range of biomedical applications, including wearable devices and scaffolds for tissue engineering.^1–6^ The water suffusing the hydrogel network enables transport of nutrients, signaling molecules, and waste products to and from cells, all contributing to improved biocompatibility.^4^ The constituent polymers and molecular design of the network renders adjustable parameters to tailor the properties of hydrogels to match the desired application and incorporate additional functionality.^3^ The advances in hydrogels for biomedical applications are apparent from a bulk material perspective, but a challenge remains: replicating the complex structure in the extracellular matrices (ECMs) or “housing” for cells. The hierarchical structure is a critical component of ECMs that synergizes with the molecular cues for successful cell differentiation, growth, proliferation, and function.^7–11^

Significant research efforts have been invested in understanding how to tailor hydrogel networks to more precisely mimic the properties of ECMs.^12–14^ In-depth studies have been carried out to understand the role of topological defects in chemically crosslinked networks.^15–18^ The range of potential architectures has expanded to encompass double network hydrogels,^19–22^ interpenetrating network hydrogels,^23^ supramolecular networks,^24,25^ and physical networks that arise as a result of water-induced self-assembly.^26–31^ Though these systems exhibit remarkable bulk properties, the primary tuning levers are molecular level changes. In contrast, ECMs exhibit hierarchical structure: multiscale ordering where each level of ordering “communicates” through the different length scales, resulting in bulk material responses. The structure and the chemistry of ECMs dictates the mechanical response, which is exemplified by the endomysium of skeletal muscles, where cooperative deformation modes span collagen fibers to aligned pores where the muscle cells reside.^11,32,33^

There are a number of preparation methods to incorporate pores into synthetic hydrogels as a means to create hierarchical structures, including cononsolvency,^34^ freeze casting,^35^ addition of porogens,^36^ and inducing phase separation,^37–39^ yet adding pores to hydrogels typically negatively impacts the mechanical properties.^40^ Recent results indicate that interconnected pores enhance the properties of hydrogels through dissipation of stress concentration.^30,34,36,37,39,41–44^ Hydrogels prepared using rapid injection processing, where a poly­(styrene)-poly­(ethylene oxide)-poly­(styrene) (SOS) triblock copolymer solution in a water miscible organic solvent is injected into a water bath to induce gelation, exhibit interconnected pores with topologically complex surfaces.^28,30^ The porous, hierarchically ordered hydrogels exhibit both nano and macroscale ordering, which is attributed to co-occurring microphase and macrophase separation processes during solvent exchange when the polymer solution is injected into the water bath. The microphase separation process where the hydrophobic end-blocks self-assemble into micelle cores at the nanoscale function as physical crosslinks bridged by the hydrophilic mid-block to form a network.^27–29,45^ At the microscale, pores are formed within the micelle network due to macrophase separation-induced solvent exchange, analogous to asymmetric membrane formation via non-solvent induced phase separation.^46^ The physical crosslinks formed during triblock copolymer microphase separation kinetically trap the macrophase separation process, resulting in the formation of macroscopic pores.

The porous hydrogels prepared using rapid injection processing exhibit drastically different mechanical properties than traditional physically crosslinked SOS triblock copolymer hydrogels without pores.^27^ These porous hydrogels exhibit extremely low moduli and tensile strength, exceptionally high extensibility, minimal hysteresis, and strain hardening behavior.^28–30^ Hydrogels with different pore sizes and structures were prepared using the same SOS triblock copolymer by varying the rapid injection processing conditions.^30^ Interestingly, as the average pore size decreased, the tensile modulus and tensile stress increased while water swelling and nanostructure remained constant.

An underlying question arose regarding the role of the bridging mid-block chains separating the micelle cores in physically crosslinked SOS hydrogels with respect to hydrogel structure and mechanical properties. In both thermodynamic (i.e., thermally annealed and the hydrated hydrogels with no pores)^27^ and non-equilibrium (i.e., prepared using rapid injection processing)^28,30^ hydrogel structures, there are looped chains where the S end-blocks of the same SOS triblock copolymer reside in the same micelle (Figure 1).^47^ Looped chains are non-elastically effective strands, reducing the modulus of the materials.^18^ Interlocking loops are elastically effective and will affect the mechanical response, but differentiating them from bridging chains is difficult.^48–50^ Under highly non-equilibrium conditions, as in rapid injection processing,^28,30^ it is difficult to estimate the fraction of hydrophilic mid-blocks bridging adjacent micelle cores. Determining the fraction of bridging and looping chains in porous, hierarchically ordered hydrogels is not trivial, as the heterogenous nature of the material is highly non-ideal and using traditional network theory is not applicable.^18^ In an effort to address the effects of non-elastically effective strands on porous, hierarchically ordered hydrogel structure and mechanical properties, we hypothesized that adding a SO diblock that is chemically identical to the SOS triblock will artificially reduce the number of elastically effective chains by adding dangling chain end defects (Figure 1).

**Figure 1:**
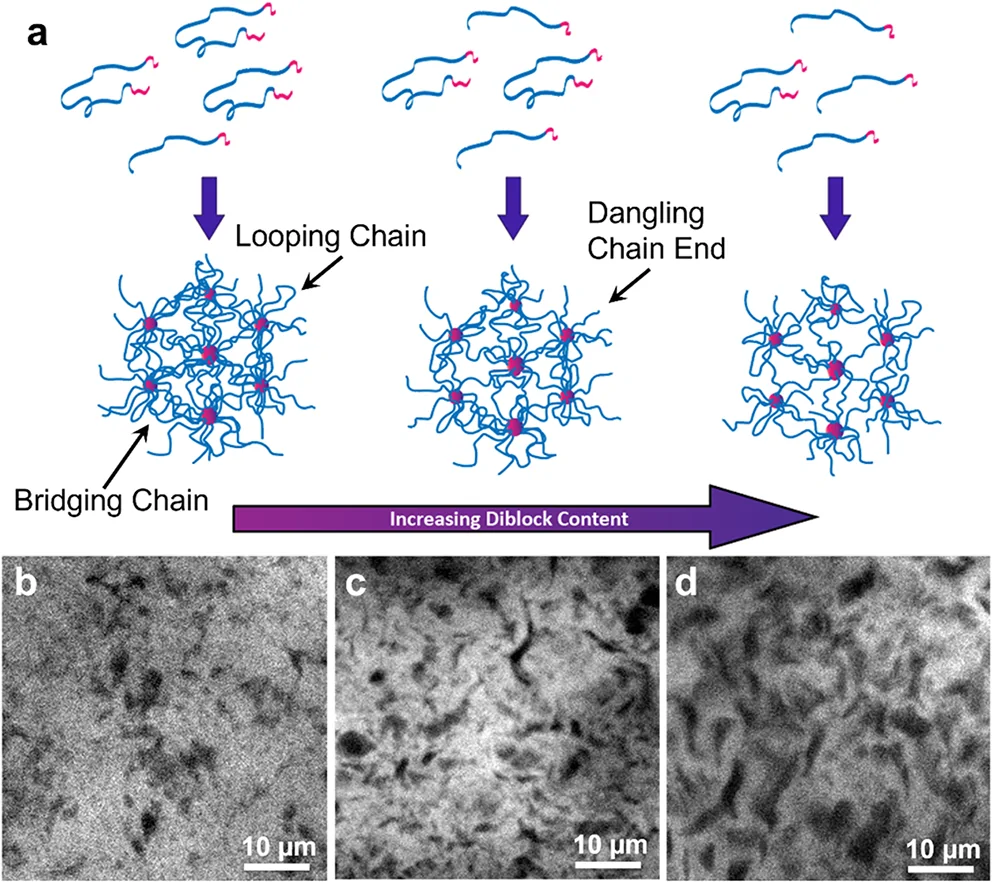
Increasing the SO diblock copolymer content results in larger macroscale pores and softer hydrogels. (a) Increasing the SO to SOS ratio results in a constant nanoscale structure of hierarchically ordered hydrogels but a drastic change in the microscale pore size. (b–d) Confocal microscope images, (b) 72 wt %, (c) 50 wt %, and (d) 25 wt % SOS triblock copolymers, highlighting the increase in pore size with increasing SO diblock copolymer content. Hydrogels were stained with 0.01 mg/mL Nile Red in DMSO overnight before confocal imaging. The highlighted regions of the confocal images show the polymer-rich domains, whereas the dark regions are the water-rich domains.

Here, we find that increasing the diblock content in the triblock copolymer leads to a significant change in the macropore morphology, as characterized by both confocal and cryogenic scanning electron microscopy (cryo-SEM) imaging. Simultaneously, the swelling ratio and pore wall thickness increased with diblock content, likely due to the increased conformational freedom of the diblock as opposed to a triblock copolymer. The mechanical properties also changed dramatically; hydrogels became softer, less tough, and fractured at lower extensions with decreasing triblock copolymer content, which is attributed to the reduced number of physical crosslinks due to diblock copolymer and larger pores. In situ confocal imaging while deforming fibers under uniaxial extension reveal that all pores align in the stretch direction at the same extension, while the larger pores prepared with low triblock content became more aligned, which is accredited to the lower modulus of the polymer-rich pore walls. Stress–strain fitting results from an additive nominal-stress constitutive model where the total stress is decomposed into pore and polymer-strand contributions enabled the determination of the tensile moduli of the pores (*E*_m_) and the load-bearing polymer-strand network (*E*_s_). Due to the multifaceted nature of the diblock dopant, it is difficult to isolate the effects of reduced bridging fraction from changes in pore size and morphology, but we clearly demonstrate that the diblock concentration is a crucial parameter that allows us to further expand the design space of these porous, hierarchically ordered hydrogels.

## Experimental Section

### Hydrogel Preparation

The poly­(styrene)-poly­(ethylene oxide)-poly­(styrene) (SOS) triblock and SO diblock copolymers used to prepare porous, hierarchically ordered hydrogels were synthesized by sequential living anionic polymerization.^28–30^ First, a 41 kg/mol S-block was synthesized, then re-initiated to form SO diblocks (Figure S1). At each stage of the synthesis process, the reaction products were precipitated, dried, and characterized via size exclusion chromatography (SEC) and proton nuclear magnetic resonance (^1^H NMR) spectroscopy (Figure S2). A portion of the SO diblock copolymer was retained for doping before coupling the SO diblock to form SOS triblock copolymer.

The synthesized triblock copolymer was determined to have a O-block volume fraction of *f*_O_ = 0.52. Due to the inherent limitations of the coupling process, some diblock remains in the triblock copolymer used in this study. The wt % of the SOS triblock, which was 72 wt %, was determined by fitting the differential refractive index (dRI) trace of the SEC with two Gaussian peaks, corresponding with the di- and triblock copolymers (Figure S3).

Hydrogel fibers were prepared by injecting a SOS triblock copolymer solution, initially in tetrahydrofuran (THF), into a reverse osmosis (RO) water bath using a syringe pump (NE-300 Infusion Syringe Pump). SOS/SO solution concentrations in THF were 14, 15, 16, 17, and 18 wt % for hydrogels containing 25, 40, 50, 60, and 72 wt % SOS, respectively. Slight heating at 50 °C for approximately 15 min was necessary to fully dissolve the polymer in THF before injection. Polymer solutions were injected into a RO water bath using a syringe pump at a speed of 1 mL/min and a needle gauge of 19 attached to a 5 mL syringe. Each hydrogel sample was prepared by injecting a 1 mL of a polymer solution into a 1 L crystallizing dish containing RO water. Gelation occurred instantaneously, resulting in fiber formation, when the THF polymer solution was injected into the RO water bath. Hydrogel fibers were retained in the original RO water bath overnight (approximately 15 h) and then moved to a new RO water bath for an additional 9 h to ensure complete solvent exchange.

Hydrogels with a given mass of triblock, the total number of chains, *N*_tot_, is given by eq 1, where *m*_*i*_, *M*_w,*i*_, and *X*_*i*_ are the mass, weight-average molecular weight, and mass fraction of species *i*, respectively.

1 $$N_{tot}=\frac{N_{AV}m_{SOS}}{M_{w,SO}}\left(1-\frac{X_{SOS}}{2}\right)$$

The number of chains in a given mass of diblock, *N*_SO_, is given in eq 2

2 $$N_{SO}=\frac{N_{AV}m_{SO}}{M_{w,SO}}$$

Thus, when doped with diblock, the new number of chains per unit mass of triblock becomes

3 $$N_{t}=\frac{N_{AV}}{M_{w,SO}}\left[m_{SOS}\left(1-\frac{X_{SOS}}{2}\right)+m_{SO}\right]$$

Equation 3 can be re-written to solve for the modified triblock wt % after doping for *X*_SOS,new_ using the new number of chains *N*_*t*,new_, and total mass *m*_*t*,mix_ (eq 4)­

4 $$X_{sos,mix}=2\left(1-\frac{(N_{t,mix}{)M}_{W,SO}}{N_{AV}{(m}_{t,mix})}\right)$$

In addition to the synthesized SOS triblock copolymer with 72 wt % triblock content, four blends of triblock and diblock copolymers were prepared at 60, 50, 40, and 25 wt % triblock using the relative mass fractions of the coupling reaction product and diblock. The trend in molecular weights was confirmed experimentally via size exclusion chromatography (Figure S4), where the increasing elution time of the samples and peak broadening signals a decrease in triblock copolymer content.

As the triblock content decreases, the total mass required to reach the set number of chains per unit volume decreases, since the diblock copolymers possess a molecular weight that is half that of the coupled triblock copolymer. The range of solution concentrations was 14–18 wt % in tetrahydrofuran (THF) for the five sample conditions, with each consecutive drop in triblock wt % lowering the initial solution concentration by approximately 1 wt %.

### Small-Angle X-ray Scattering (SAXS)

SAXS measurements were conducted at the Brookhaven National Laboratory, National Synchrotron Light Source II 11-CMS beamline. Samples were exposed for 3 s using a 13.5 keV beam with a wavelength of 0.9184 Å. A sample-to-detector distance of 10 m was used to access the *q*-range 0.0044–0.03 Å^–1^. 2D scattering patterns were obtained using a Pilatus 2M detector. A measurement of an empty position was used to subtract the background from the hydrogel and converted to a 1D plot of intensity versus *q*. The background subtraction and conversion were performed for every sample measured at the beamline using the program SciAnalysis. Where a disordered spherical morphology was evident in the scattering data, Igor Pro equipped with the IRENA package downloaded from Argonne National Lab^51^ was used to fit the measurements with theoretical predictions. The data was loaded into IRENA and fit with a spheroid form factor and a hard sphere structure factor derived from the Percus–Yevick approximation.

### Cryogenic Scanning Electron Microscopy (Cryo-SEM)

Imaging of the porous microstructure was achieved via cryo-scanning electron microscopy (Cryo-SEM). Hydrogel fibers were cut perpendicular to their length vitrified, and microtomed until the height of the sample had been reduced by at least a half millimeter to ensure an accurate cross-section. They were then transferred into a Zeiss SIGMA VP-FESEM antechamber at −90 °C and held for 15 min to allow partial sublimation of the surface of the sample. The samples were sputter coated with a gold–palladium alloy, then transferred into the primary chamber for imaging at −125°C. Samples were imaged at a beam energy of 1–1.5 kV.

### Confocal Imaging

Glass coverslips (No. 1) were first cleaned in sequence with soap and water, acetone, and deionized water, then dried with house air. The clean coverslip was set up on a Leica DMI 4000B microscope sample stage. Confocal fluorescence imaging was performed on hydrogel samples >2.6 cm in length, stained with Nile Red. The dye solution was prepared by first dissolving 0.1 mg/mL Nile Red in DMSO and then diluting it further to 0.01 mg/mL in DI water. Samples were aligned lengthwise on the clean coverslip and clamped on both ends. A 100× objective was used to focus on the surface and 550–700 nm excitation range of laser was used to excite the dye. Images were acquired at the initial length of 2.6 cm (λ = 1.00), stretched and reclamped to 3.4 cm (λ = 1.31), and finally after further extension to 4.2 cm (λ = 1.62). 10 images were taken at each surface and averaged for better signal quality.

Pore size, shape and orientation were quantified from the fluorescence images using Fiji (ImageJ). For all samples, one fiber was analyzed, and a z-stack of 50 images, 109 × 109 μm^2^, separated by 0.2 μm was analyzed. Therefore, the error bars reflect the variability across images within a single sample. Pore sizes were quantified by fitting a circle to the widest region of each pore and measuring the circle diameter using the built-in measurement tools in Fiji. The resulting pore diameters were then averaged to obtain the mean pore size. Circularity was used as a shape descriptor to report how closely each segmented pore approximates to a perfect circle, and orientation was defined as the angle of the pore’s major axis of the best-fit ellipse relative to the *x*-axis. To isolate pores from the surrounding gel, grayscale images were thresholded to generate a binary mask. Dark regions with low intensity correspond to pores, as they are untagged and lack fluorescent signal, whereas bright regions with high comparative intensity correspond to the dye-labeled gel. Pixels below the selected intensity threshold were classified as pores, and all remaining pixels were classified as gel.

For each pore object in the binary mask, the circularity *C* was calculated from the measured area *A* and perimeter *P*

5 $$C=\frac{4\pi A}{P^{2}}$$

A value of *C* = 1 corresponds to a perfect circle, while values approaching 0 indicate increasingly elongated or irregular pore geometries. The orientation of each pore was obtained simultaneously as the angle of the major axis of the fitted ellipse, providing a measure of pore alignment relative to the horizontal axis.

Pore orientation histograms were analyzed using axial circular statistics, where (0°) and (180°) represent the same orientation. For each strain state, histogram counts were normalized to probabilities (*p*). For each strain state, the orientation parameter relative to the stretch axis was calculated as *S* = ∑_i_ *p*_i_cos­[2­(θ_i_ – θ_s_)], where *p*_i_ is the normalized probability in angular bin i. The stretch-induced orientation was then normalized by the initial orientation as *S*_norm_ = (*S*_ϵ_ – *S*_0_)/(1 – *S*_0_), representing the fraction of remaining possible alignment achieved during stretching.

Pore circularity was quantified from binned circularity histograms at each stretch ratio λ. For each condition, histogram counts were normalized to probabilities, where *p*_ic_ is the probability of bin i and *C*_i_ is the circularity bin center. The weighted mean circularity was calculated as, *C̅* = ∑*p*_ic_ *C*_i_. Relative circularity was then calculated by normalizing each condition to the initial state, $C_{normalized}=\frac{\mathrm{C̅}\left(\lambda \right)}{{\mathrm{C̅}}_{0}}$ . Values greater than 1 indicate increased pore circularity relative to the initial state, while values less than 1 indicate reduced circularity.

### Uniaxial Extension

The mechanical properties were characterized via uniaxial extension testing on MTS Criterion load frame with a 10 N force transducer. The hydrogels were secured with spring-action tensile grips, preventing hydrogel slip during extension, and strained at a rate of 3/min. Hydrogel fiber cross-sectional areas were determined by slicing the fibers into smaller pieces and placing them on a glass slide and imaged using an Olympus 100 optical microscope.^30^ The areas of the cross sections were calculated using ImageJ.

### Nominal-Stress Constitutive Model

The stress–strain curves were modeled using an additive nominal-stress constitutive model in which the total stress is decomposed into pore and polymer-strand contributions

6 $$\sigma \left(\lambda \right)=\sigma_{p}\left(\lambda \right)+\sigma_{s}\left(\lambda \right)$$

where σ is the nominal-stress and λ is extension ratio. The decomposition into pore and polymer-strand contributions is motivated by confocal microscopy observations showing that deformation is first accommodated by pore closure, followed by polymer-strand stretching after the pores are fully closed.

The pore contribution is described using a dilute isolated-pore approximation.^52–54^ The porous hydrogel is treated as an elastic matrix containing an evolving pore volume fraction ϕ­(λ). The effective shear modulus is written as

7 $$E_{\mathrm{eff}}\left(\lambda \right)=E_{m}\left[1-a\phi \left(\lambda \right)\right]$$

where *E*_m_ is the shear modulus of the nonporous matrix, *a* is a pore-softening coefficient that depends on pore shape and geometry, and ϕ­(λ) is the current pore volume fraction. The corresponding nominal pore stress is

8 $$\sigma_{p}\left(\lambda \right)=\frac{E_{m}}{3}\left[1-a\phi \left(\lambda \right)\right]\left(\lambda -\lambda^{-2}\right)$$

Pore closure is described using a scalar closure variable *c*_p_(*λ*), where

9 $$c_{p}=0$$

corresponds to open pores and

10 $$c_{p}=1$$

corresponds to complete pore closure. From microscopy, pores begin to close at λ_onset_ and we assume that at λ_act_ they are “closed” and no longer contribute to the stress response. We assume that closure is smooth and not abrupt and define a normalized closure coordinate as

11 $$x=\frac{\lambda -\lambda_{\mathrm{onset}}}{\lambda_{\mathrm{act}}-\lambda_{\mathrm{onset}}}$$

with *x* bounded between 0 and 1. The closure variable is then prescribed using a smoothstep function

12 $$c_{p}\left(\lambda \right)=3x^{2}-2x^{3}$$

The pore volume fraction evolves according to

13 $$\phi \left(\lambda \right)=\phi_{0}\left[1-c_{p}\left(\lambda \right)\right]$$

where ϕ_0_ is the initial pore volume fraction. Thus, ϕ = ϕ_0_ before pore closure begins, and ϕ = 0 once λ ≥ λ_act_.

The polymer network is assumed to become load-bearing only after complete pore closure. Therefore, the polymer contribution is activated sharply at λ_act_. The polymer contribution is written as

14 $$\sigma_{s}\left(\lambda \right)=0,\lambda <\lambda_{\mathrm{act}}$$

and

15 $$\sigma_{s}\left(\lambda \right)=\frac{E_{s}}{3}\frac{\lambda_{s}-\lambda_{s}^{-2}}{1-\frac{I_{1,s}-3}{J_{m}}},\lambda \geq \lambda_{\mathrm{act}}$$

where *E*_s_ is the tensile modulus of the load-bearing polymer-strand network, *J*_m_ is the finite-extensibility parameter, and

16 $$I_{1,s}=\lambda_{s}^{2}+2\lambda_{s}^{-1}$$

The effective strand stretch is defined relative to the fully closed-pore state^55,56^

17 $$\lambda_{s}=\frac{\lambda }{\lambda_{\mathrm{act}}},\lambda \geq \lambda_{\mathrm{act}}$$

This definition assumes that polymer strands do not carry appreciable load before pore closure and that deformation accumulated after *λ*_act_ is transmitted directly to the polymer network.

The full nominal-stress model is therefore

18 $$\left(\lambda \right)=\frac{E_{m}}{3}\left[1-a\phi \left(\lambda \right)\right]\left(\lambda -\lambda^{-2}\right)+\frac{H\left(\lambda -\lambda_{\mathrm{act}}\right)E_{s}}{3}\frac{\lambda_{s}-\lambda_{s}^{-2}}{1-\frac{\lambda_{s}^{2}+2\lambda_{s}^{-1}-3}{J_{m}}}$$

where *H*(λ – λ_act_) is the Heaviside function, equal to zero before pore closure and one after pore closure. This model captures the experimentally observed sequence of pore closure followed by polymer-strand stretching and finite-extensibility strain hardening.

## Results and Discussion

### Characterization of the Hydrogel Nanostructure

SOS hydrogel fibers were prepared using a non-equilibrium process, rapid injection processing, where a polymer solution in tetrahydrofuran (THF) is injected into water via a syringe pump.^30^ Upon injection of the SOS solution into water, solvent exchange between water and THF led to hydrogel fiber formation. The SOS triblock used here exhibited a total number-average molecular weight (*M*_n_) of 136 kg/mol (with S end-blocks of 41 kg/mol) and an O-block volume fraction of 0.52. Size exclusion chromatography (SEC) and proton nuclear magnetic resonance (^1^H NMR) spectroscopy used for characterizing the molecular weight and block volume fraction are shown in Figures S1 and S2. The SOS triblock had 72 wt % triblock content with 28 wt % being uncoupled SO diblock copolymer (Figure S3). The impact of SO diblock copolymer on hydrogel structure and properties was assessed by doping in SO diblock to the SOS triblock using four different blends: 60, 50, 40, and 25 wt % triblock.

Using a singular initial solution concentration for samples containing 72, 60, 50, 40, and 25 wt % triblock would result in solutions with drastically different viscosities, potentially masking the effect of decreasing the number of elastically effective strands through diblock doping. Therefore, we prepared two different sample series: (1) equal number of triblock/diblock chains per volume in the pre-injection solution and (2) different initial 72 wt % SOS triblock copolymer solution concentrations to compare to the hydrogels prepared at equal number of chains per volume. Thus, SOS/SO solution concentrations at constant number of chains per volume were in the 14–18 wt % range, with each consecutive decrease in triblock content lowering the initial solution concentration by approximately 1 wt % (Figure S4). The calculations for preparing SOS THF solutions with the same number of chains are described in the Experimental Section. To verify the effects on mechanical properties are not due to initial solution concentration, we prepared hydrogels using the 72 wt % SOS triblock copolymer composition at initial solution concentrations matching those with reduced SOS triblock content. Thus, we were able to directly analyze the impact of the decreasing number of elastically effective strands (i.e., adding SO relative to SOS) in a controlled manner, while comparing the pore structure and mechanical properties of hydrogels prepared using 72 wt % SOS triblock at different solution concentrations.

All prepared hydrogels exhibited greater than 0.90 water content by weight (Figure 2a), with increased water swelling with increasing SO diblock copolymer content. Furthermore, the diameter of the fibers increases with increasing diblock content after polymer solution injection and solvent exchange. A comparison of the undoped triblock copolymers at the same initial polymer solution concentration as compared to the copolymer blend shows the swelling ratios of hydrogels made from the copolymer blend are significantly higher (Figure S5). We hypothesize that the cause of the increase in swelling ratio with diblock content is due to changes in the porous microstructure and/or an increase in the degree of freedom of segmental movement of the SO dangling ends, which would maximize the number of water molecules that could be accommodated within the network.^57^

**Figure 2:**
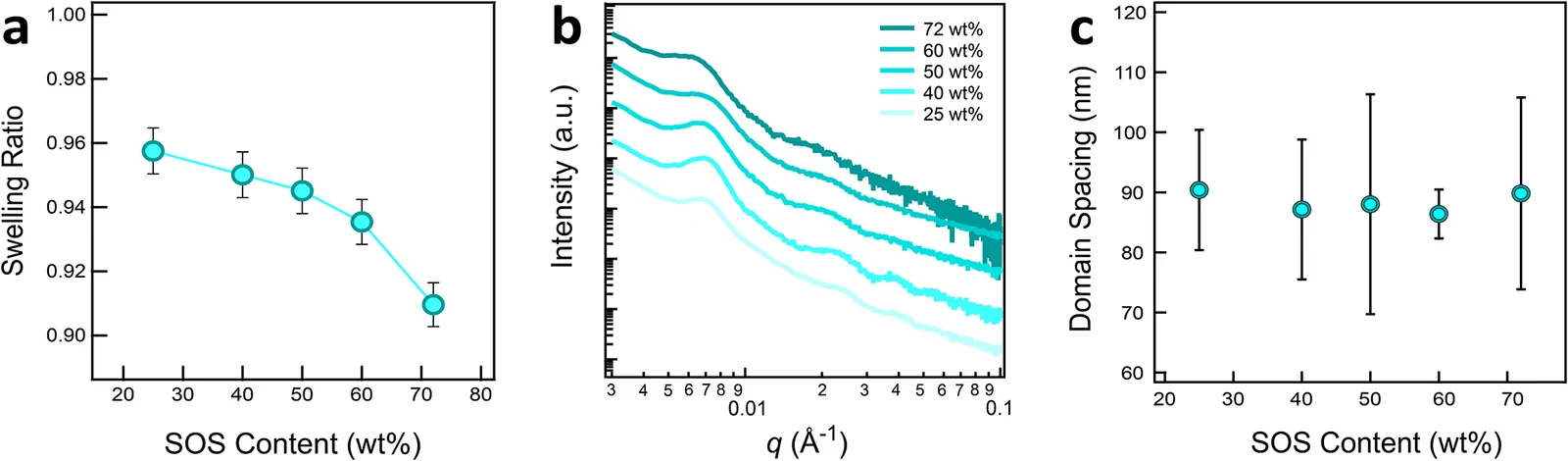
Water content and nanoscale characterization of hydrogels prepared with different SOS triblock copolymer content. (a) The degree of water swelling increases with increasing SO diblock content. (b) 1D SAXS data for hydrogels prepared with different SOS content showing that the primary scattering peak associated with the center-to-center micelle spacing remains constant. (c) Domain spacing determined from SAXS measurements remain constant and are within error as the number of chains per mL is held constant for rapid injection processing.

Small-angle X-ray scattering (SAXS) performed at the Brookhaven National Laboratory, National Synchrotron Light Source II 11-CMS beamline was used to probe the nanoscale micelle structure. All hydrogel fibers exhibited a disordered sphere morphology with a near constant *q*-value of the primary scattering peak that is associated with the micelle center-to-center distance (Figure 2b). Reported domain spacings in Figure 2c were determined by fitting the 1D SAXS plots shown in Figure 2b with a spheroid form factor and a hard sphere structure factor derived from the Percus–Yevick approximation using Igor Pro equipped with the IRENA package downloaded from Argonne National Lab (Figure S6).^51^ The domain spacing remained constant as the diblock copolymer doping agent increased. Previous work using a lower S-block molecular weight and a high *f*_O_ (e.g., *M*_n,PS_ = 13 kg/mol and *f*_O_ = 0.90)^30^ showed a consistent domain spacing with concentration, which is reproduced here in triblock copolymers with a higher S-block molecular weight and a lower *f*_O_. In all, prepared hydrogel samples exhibit similar nanoscale structures when the number of chains per mL is held constant for rapid injection processing.

### Influence of Diblock Content on Hydrogel Microstructure

Cryo-SEM micrographs of the hydrogels prepared using 72, 50, and 25 wt % SOS/SO blends (Figure 3a–c) show distinct changes in pore morphology. For hydrogels made from 72 wt % SOS triblock copolymer, the microstructure of the cross-section is uniform, with a monomodal pore size distribution and an average pore diameter of 5 μm (Figure 3a). At 50 wt % triblock, larger pores appear around the perimeter of the hydrogel fiber cross-section (Figure 3b), and at 25 wt %, the fibers exhibit a coaxial core-shell morphology that was previously observed only in hydrogels that were made from *N*,*N*–dimethylformamide (DMF) (Figure 3c).^30^ Though the underlying forces driving this transition are yet unknown, we hypothesize the increase in diblock content delays physical crosslinking due to fewer bridging chains, allowing for macrophase separation to occur over a longer time period before becoming kinetically trapped. Fundamental studies of the macrophase separation process and the impacts on hierarchical structure are ongoing and will be reported on in future work. Interestingly, the shell of the fibers, which was smooth in all previously imaged samples, is porous in hydrogels produced from 25 wt % triblock copolymer blends (Figure 3c). Again, the porous fiber shell is likely due to the coarsening of the macrophase separation process before being kinetically trapped due to bridging chains. Finally, cryo-SEM images show that the pore walls increase in thickness with increasing SO diblock copolymer content (Figure 3d), which is likely correlated with the increase in swelling ratio.

**Figure 3:**
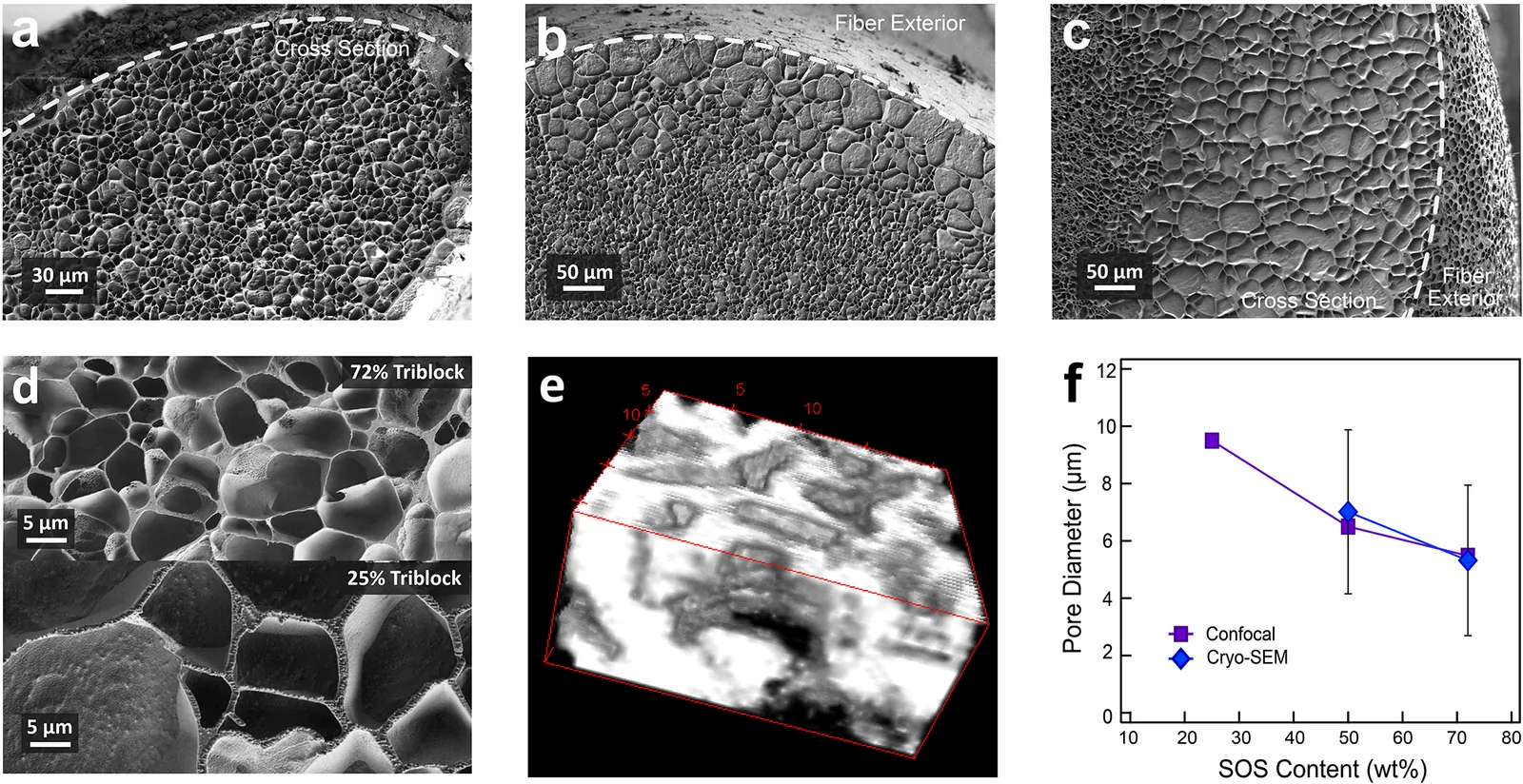
Microstructural analysis of the hydrogel pores using cryo-SEM and confocal microscopy. Cryo-SEM micrographs of hydrogels containing (a) 72 wt %, (b) 50 wt %, and (c) 25 wt % SOS triblock copolymer. Dashed white lines in (a–c) delineate the fiber cross-section from the exterior surface of the fiber. The hydrogels exhibit a change in pore microstructure with increasing diblock content. At 72 wt % triblock copolymer, there is a monomodal distribution of small pores. Hydrogel fibers exhibit a coaxial core-shell morphology at 25 wt % triblock copolymer where larger pores reside at the fiber shell and small pores in the core. (d) Cryo-SEM micrographs show how pore wall thickness increases as diblock content increases when going from 72 to 25 wt % triblock copolymer. (e) 3D rendering of a hydrogel prepared with 25 wt % triblock copolymer. The pore walls are highlighted, and the pores are void. The values shown in red indicate the length scale in μm. (f) A plot of pore diameter versus triblock copolymer content. Pore diameters were determined from both cryo-SEM and confocal micrographs.

Pore diameters for hydrogels were determined from confocal microscopy and cryo-SEM and agree with each other. A combination of cryo-SEM and confocal microscopy were used to characterize the porous microstructure to ensure that freezing artifacts during cryo-SEM hydrogel preparation do not lead to mischaracterization and confocal imaging enables in situ tensile measurements to investigate pore deformation. Confocal microscopy was performed on hydrogel samples >2.6 cm in length, stained with 0.01 mg/mL Nile Red in DMSO overnight (Figure 1b–d). Nile Red stains the hydrophobic domains, highlighting the polymer-rich pore walls. The average pore diameter decreases with increasing triblock content (Figures 3e and 1b–d). The 3D rendered confocal image in Figure 3e clearly shows the pore structure of a hydrogel prepared with 25 wt % triblock copolymer. The average pore diameter increases from 6 μm to 10 μm with decreasing SOS content from 72 wt % to 25 wt % (Figure 3f). Determining the average pore diameter from cryo-SEM micrographs for the 25 wt % triblock copolymer was difficult due to the bimodal pore distribution. Thus, the reported pore diameter in Figure 3f was determined from confocal imaging only.

In situ confocal imaging was used to quantify how pore shape and orientation evolve with deformation for hydrogel at triblock concentrations of 72, 50, and 25 wt % and extension ratios (λ) 1.00, 1.31, 1.62 (Figure 4). The 3D reconstruction of the pores shown in Figure 3e highlights that the pores are irregular and interconnected, thus further analysis is needed to understand the impact of pore microstructure on the mechanical properties. Figure 4 shows that pore circularity (*C*) and orientation angle (°) depend on both the triblock concentration and *λ*. A value of *C* = 1 corresponds to a perfect circle, while values approaching 0 indicate increasingly elongated or irregular pore geometries. Overall, unstretched hydrogels (e.g., 72, 50, and 25 wt %) exhibit irregular pores that are elongated with *C* ≈ 0.2. Furthermore, all hydrogels show pore orientation in the stretch direction, which is consistent with previous results.^30^

**Figure 4:**
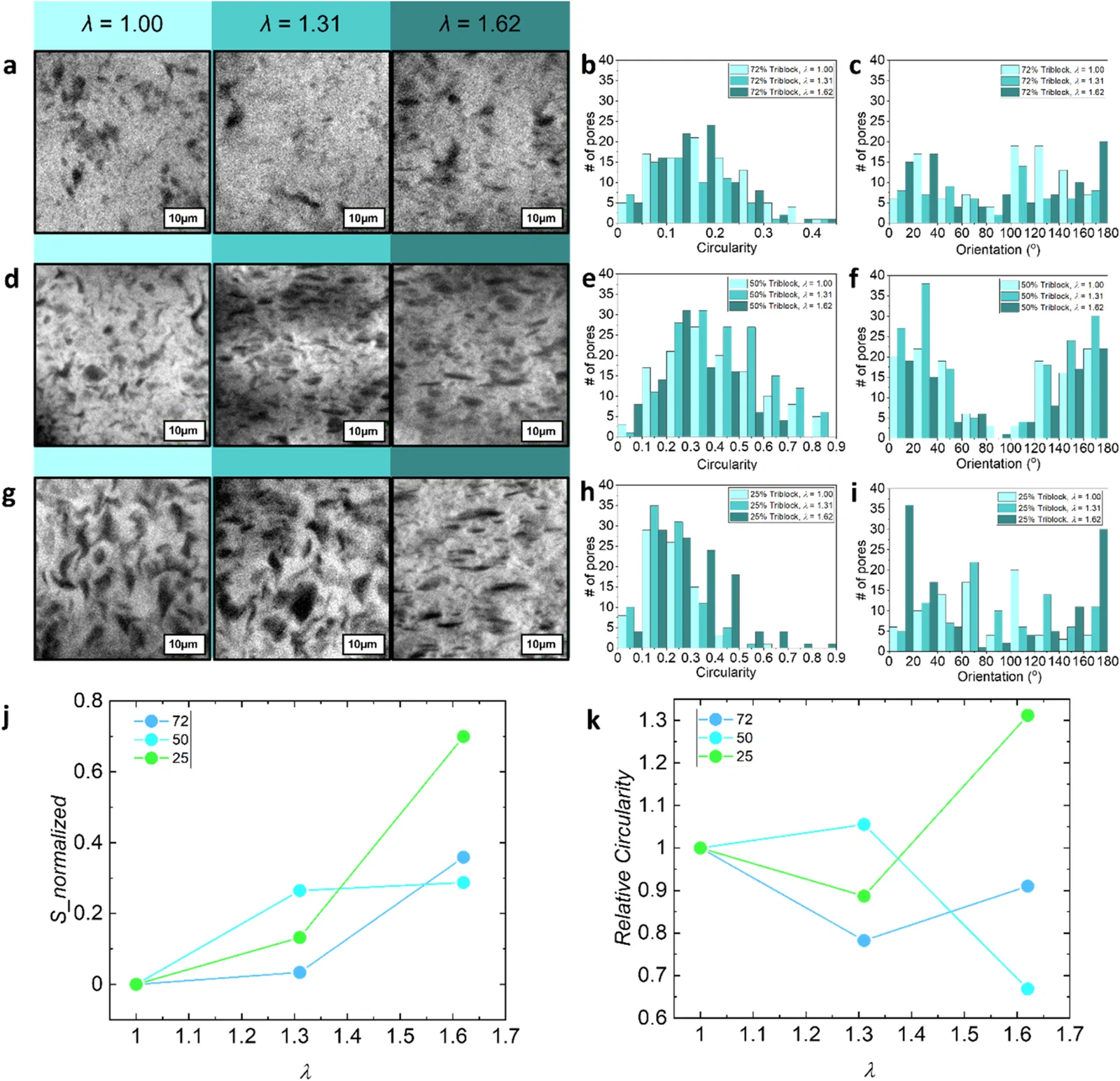
Confocal micrographs showing hydrogel pore microstructure at extension ratios λ = 1 (light blue), λ = 1.31 (darker blue) and λ = 1.62 (dark blue). (a) Confocal images of a hydrogel containing 72 wt % triblock. (b, c) Analysis of the (b) circularity and (c) orientation angle as a function of λ. (d) Hydrogel containing 50 wt % triblock. (e, f) Analysis of the (e) circularity and (f) orientation angle as a function of λ. (g) Hydrogel containing 25 wt % triblock. h, (i) Analysis of the (h) circularity and (i) orientation angle as a function of λ. 0/180° for all three samples are in the stretching direction. (j) Relative pore alignment for each gel as a function of the stretch ratio. (k) Relative circularity from the mean as a function of λ.

Pore circularity and orientation trends are clearly visualized in the histograms of Figure 4. Hydrogels with triblock concentration of 72 wt % (Figure 4a–c) exhibit pore orientation as a function of deformation at λ = 1.31 (Figure 4b,c). For a triblock concentration of 50 wt % (Figure 4d–f), the orientation in the stretching direction increases at a lower deformation, and pores are mostly aligned when λ = 1.31. At 25% triblock copolymer (Figure 4g–i), the pores are large and irregular in shape and the orientation increases in the stretching direction as a function of deformation. While the circularity remains unchanged, the orientation goes from random (light blue) to strongly oriented when λ = 1.62 in the 0/180° direction which is the axis of deformation (dark blue) (Figure 4h,i). Both the relative pore alignment and circularity from the mean for each gel as a function of the stretch ratio is shown in Figure 4j,k.

### Mechanical Properties of Hydrogel Fibers

Triblock content has an extreme effect on the mechanical properties of the hydrogels as determined from uniaxial extension measurements (Figure 5a). There is a significant drop in the Young’s modulus, ultimate tensile strength, toughness, and elongation ratio at break with decreasing triblock content (Figure 5a,b), which is correlated with an increase in pore size and increase pore orientation under strain. A reduction of elastically effective chains reduces the Young’s modulus of the materials, and it is likely that large, oriented pores, coupled with a reduction in the number of elastically effective strands, result in much more rapid fracture at low triblock contents. The results in Figure 5a,b indicate that the differences in triblock content of 10 wt % or less generate significant differences in hydrogel mechanical properties.

**Figure 5:**
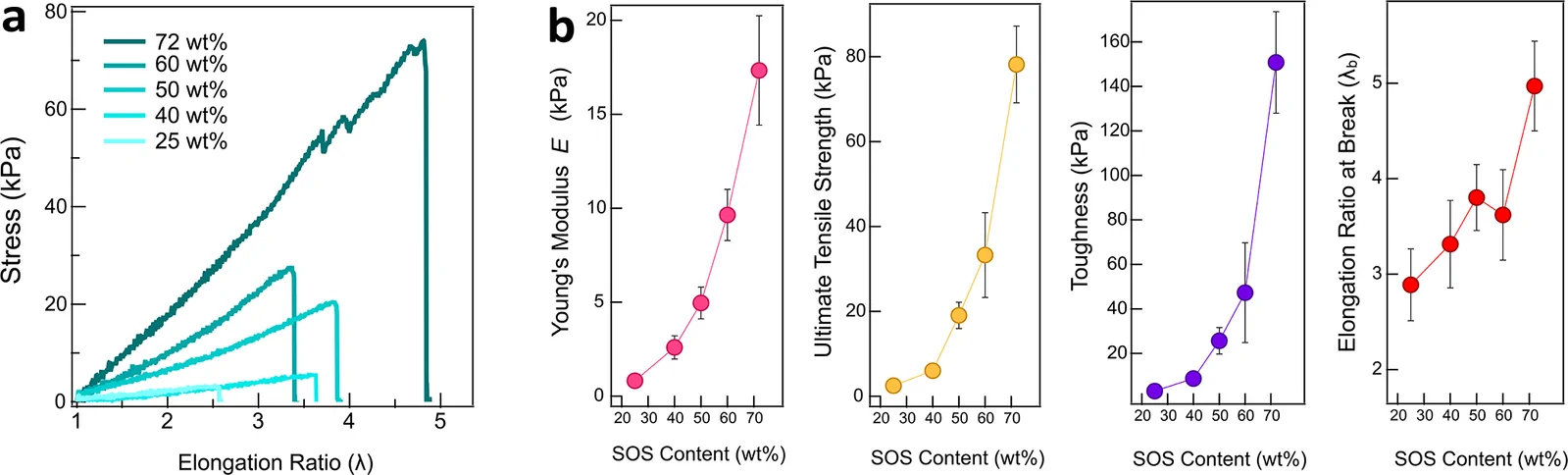
Mechanical properties of hydrogels with different triblock/diblock copolymer ratios. (a) Stress–strain curves from uniaxial extension measurements showing a decrease in the mechanical properties with increasing diblock copolymer content. (b) Trends in Young’s Modulus, ultimate tensile strength, toughness, and elongation at break with respect to triblock copolymer wt % determined from stress–strain curves (Figure S7).

The experimental data is consistent with a mechanical model that incorporates pore-mediated and polymer-strand contributions described by eq 18 and shown in Figure 6.^52–56^ The initial pore volume fraction, ϕ_0_, was estimated directly from confocal microscopy images (Figure 4) by segmenting the pore phase from the polymer-rich phase. For each image, the pore area fraction was calculated as

19 $$\phi_{A}=\frac{A_{\mathrm{pore}}}{A_{\mathrm{total}}}$$

**Figure 6:**
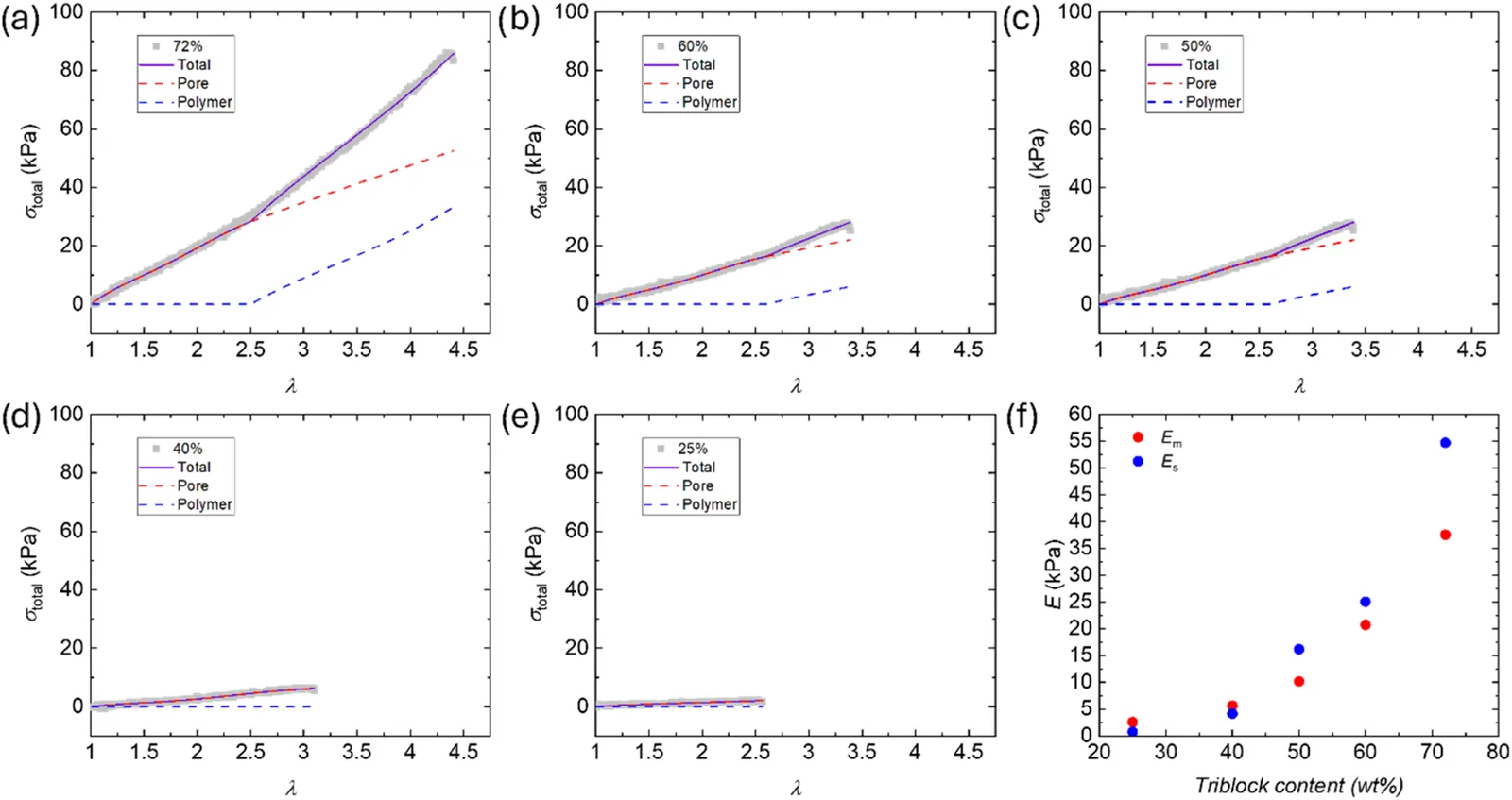
Fitted stress–strain curves showing both the pore contribution (red) and the polymer contribution (blue) for the porous hydrogels with triblock content ranging from 72 wt % to 25 wt % (a–e). (f) Both the tensile modulus and the hydrogel modulus increase as a function of triblock content.

where *A*_pore_ is the segmented pore area and *A*_total_ is the total image area. Assuming the confocal slices are representative of the bulk pore structure, the pore volume fraction was approximated as

20 $$\phi_{0}\approx \phi_{A}$$

The volume fraction increases from 0.49 to 0.58 as the triblock wt % decreases. The pore closure stretches were also estimated from confocal images. The stretch at which visible pore deformation first began was assigned as λ_onset_, while the stretch at which the pores were fully closed was estimated as λ_act_ from the stress–strain curve. For example, for the 72% sample, the pores are already physically deformed at extension ratio of 1.31, so λ_onset_ was chosen to be 1.31. This is evidenced from both the images and from the orientation parameter in Figure 4j. The 50% sample was similar. For the 25% sample, the deformation is not significant until λ is 1.62, so λ_onset_ was chosen to be that value. λ_act_ was a fitting parameter for the model, generally equal to λ_onset_ + 1. For the data that did not have imaging available (the 40 and 60% samples), an average of the λ_onset_ was used for closest compositions. For example, for the 40% data, λ_onset_ was an average of the 25% and the 50% data. These values were then used as fixed inputs in the constitutive model, rather than treated as fitting parameters.

The finite-extensibility parameter, *J*_m_, controls the onset of strain hardening in the polymer-strand contribution, was fitted using the sample with the highest polymer volume fraction, where the polymer network contribution was most pronounced. It was found to be equal to 5, which implies that the chains are close to extended in the gel. The resulting *J*_m_ value was then held constant for the remaining samples. This approach assumes that the molecular extensibility of the load-bearing O-block strands is the same across formulations, while the effective moduli of the pore matrix and polymer network vary with composition.

The fitted results show that both the pore-associated modulus, *E*_m_, and the polymer-strand modulus, *E*_s_, increase with increasing SOS content (Figure 6). At low SOS fractions, deformation is dominated by pore closure, and the polymer network is too soft to support large stresses after pore collapse. At higher SOS fractions, the polymer network contributes more strongly after pore closure, producing a larger post-activation stress and more pronounced strain hardening.

The pore-softening coefficient *a* accounts for the reduction in effective modulus caused by the pores and depends on the pore radius, orientation, and shape. Across the 40–72 wt % triblock samples, the pore-softening coefficient remains approximately constant at 0.5, whereas the 25 wt % sample exhibits a substantially lower value of 0.12. Relative to the characteristic value of the other formulations, this corresponds to an effective morphology factor of 0.12/0.5 = 0.24. The magnitude of this deviation is consistent with the distinct morphology of the 25 wt % sample, which contains large, irregular, interconnected pores and a heterogeneous core–shell pore structure. These features depart strongly from the dilute, approximately isolated-pore geometry assumed in the constitutive model. Therefore, the fitted parameter a for this composition is interpreted as an effective geometric parameter rather than a universal pore-softening coefficient.

The combination of SAXS, confocal, and mechanical properties clearly demonstrate that the diblock concentration is a crucial parameter in both the macropore structure and the deformation response. Reducing the number of bridging chains through the addition of diblock copolymer is expected to lead to softer hydrogels, which is consistent with the reduction in mechanical properties. Although the focus of the current work is on changing the bridging SOS fraction by doping in SO diblock copolymers, interlocking loops are possible and will affect the mechanical response, but quantifying the fraction is difficult.^48–50^ The effect of the macropore structure affects the deformation response,^30^ and confocal imaging confirms that pores deform more at lower triblock content while deformation starts earlier for higher triblock content. This is consistent with the reported mechanical response: larger pores lead to a lower Young’s modulus and strain hardening starts sooner for higher triblock content since the strain on individual chains begins at lower extension ratios. Thus, both the number of elastically effective chains and macropore structure will control the mechanical properties, but it is difficult to fully isolate the effects of bridging chains and macropore size.

### Effect of Initial Polymer Solution Concentration

Previous work has demonstrated solution concentration impacts the mechanical properties of samples produced using THF due to the change in the macropore size.^30^ Specifically, larger pores form at lower pre-injection polymer solution concentrations, leading lead to softer hydrogels,^30^ which is consistent with natural cellular materials.^58^ To correctly attribute the trends in mechanical performance with decreasing triblock content, as shown in Figure 5, rather than the concentration of the initial pre-injection solution, equivalent solution concentrations using only the 72 wt % triblock copolymer sample were prepared. Figure 7 compares the mechanical properties of the hydrogels comprised of the 72 wt % triblock copolymer sample at the same concentration of the triblock/diblock copolymer hydrogel samples (SOS/SO). The results shown in Figure 7 confirm that the mechanical properties of the hydrogels with increasing SO content are not caused by decreases in overall polymer solution concentration. Specifically, the trends in Young’s modulus, ultimate tensile strength, toughness, and elongation ratio at break with respect to initial polymer solution concentration for hydrogels prepared using the 72 wt % triblock copolymer sample and the SOS/SO mixtures vary significantly. Though the Young’s modulus and ultimate tensile strength are more strongly affected by initial solution concentration than elongation ratio at break and toughness, all four properties display less change in mechanical properties with respect to concentration as opposed to when the triblock content is also decreasing (Figure 7).

**Figure 7:**
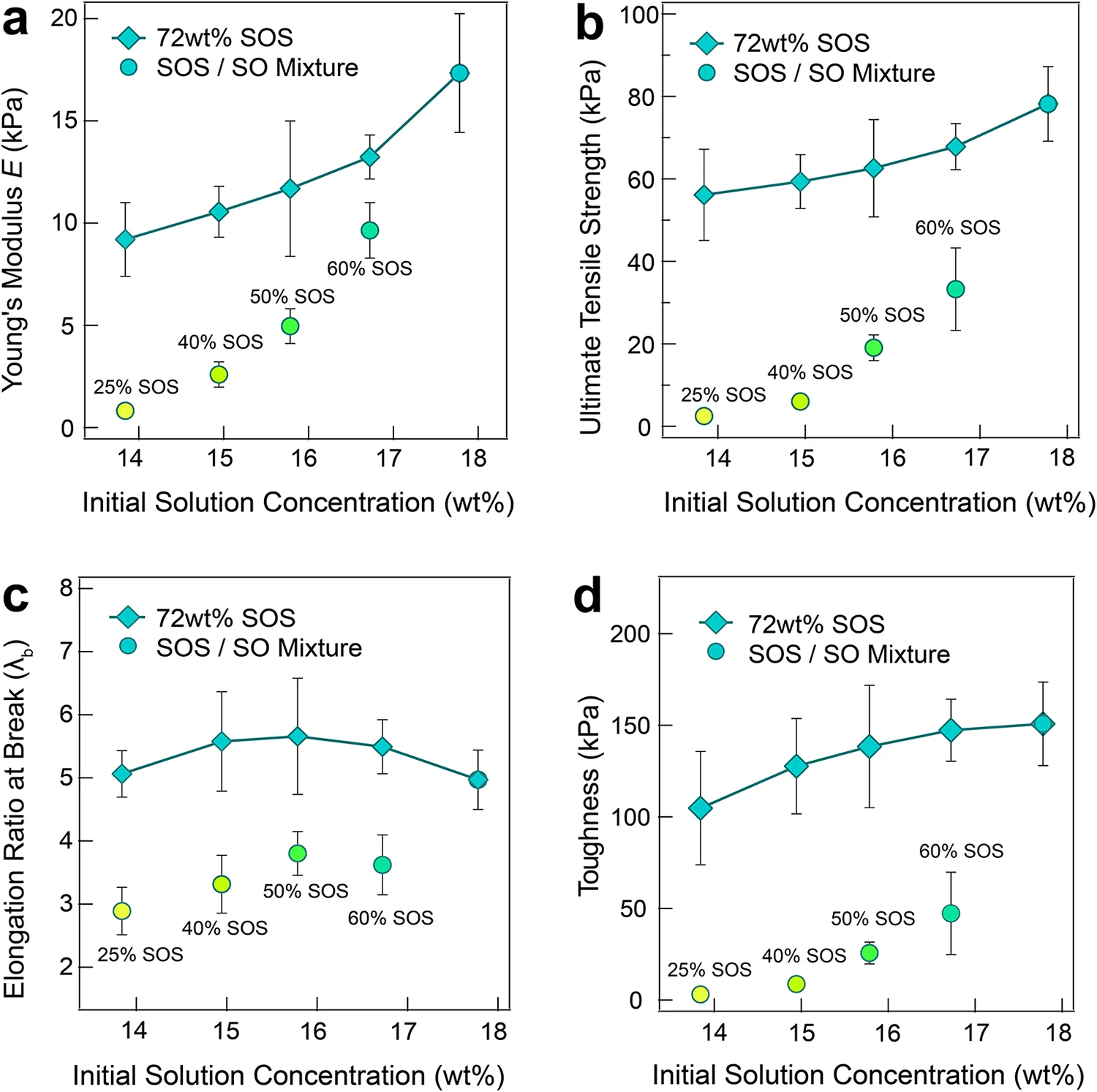
Comparison of hydrogel mechanical properties at different initial polymer solution concentrations when the number of SOS/SO chains are the same (diamond) and different (circle). Plotted values for (a) Young’s modulus, (b) ultimate tensile strength, (c) elongation ratio at break, and (d) toughness determined from stress–strain curves.

## Conclusions

Here, we clearly demonstrate that the diblock concentration is a crucial parameter that further expands the design space of porous, hierarchically ordered hydrogels. These hydrogels demonstrate mechanical properties that are tied to the hierarchical structure (molecular to macroscale), and doping SOS triblock copolymers with SO diblock copolymers is a powerful method of modifying mechanical properties and deformation responses of porous, hierarchically ordered hydrogels while maintaining the same chemical composition. There are a number of methodologies to toughen hydrogels, such as the introduction of a second network or additional chemical functionality,^35,59^ but the ability to soften hydrogels without changing the chemical composition and preserving the relative fraction of the hydrophilic and hydrophobic moieties in the polymer offers unique ways to control hydrogel properties.

The interplay between softening the polymer network through SO addition while simultaneously tuning the pore structure has a direct impact on the deformation response. In situ confocal imaging highlights how pore alignment in the stretch direction is impacted by the modulus of the polymer network. It is hypothesized that adding SO diblock copolymers to the SOS hydrogels reduces the bridging chain fraction, but the direct relationship between bridging SOS chain fraction and pore morphology and size are still open questions.

## Supplementary Material



## Data Availability

The raw data supporting the findings of this study are available at Data Commons at Penn State at (10.26208/6ZXF-5Y82)

## Notes

^1^H NMR, Small-angle X-ray scattering, mechanical testing (PDF)
