# Supplementary material for: Diblock/Triblock Composition Controls Pore Structure and Mechanical Response in Hierarchically Ordered Hydrogels
Source: ACS Appl Polym Mater. 2026 Sep 7;8(18):16647–58. doi: 10.1021/acsapm.6c02944 (PMC13621640; doi:10.1021/acsapm.6c02944)
Supplement: Supplementary file 1 [file ap6c02944_si_001.pdf]

Supporting Information

Diblock/Triblock Composition Controls Pore Structure and Mechanical Response in Hierarchically Ordered Hydrogels

Elisabeth C. Lloyd,[a] Sujata Dhakal,[b] Sathika B. Arachchige,[c] Mykyta Dementyev,[a] Shubhra Goel,[a] Svetlana Morozova,[b,]* Robert J. Hickey[a,d,]*

[a]Materials Science and Engineering, The Pennsylvania State University, University Park, PA 16802, USA
[b]Macromolecular Science and Engineering, Case Western Reserve University, Cleveland, OH 44106, USA
[c]Department of Chemistry, The Pennsylvania State University, University Park, PA 16802, USA
[d]Materials Research Institute, The Pennsylvania State University, University Park, PA 16802, USA
*Corresponding authors: rjh64@psu.edu & sam381@case.edu

Table of Contents

- **Figure S1. Size exclusion chromatography (SEC) of polymers**
- **Table S1. Molecular characteristics of the synthesized polymers**
- **Figure S2. ^{1}H NMR spectrum of the SOS triblock copolymer**
- **Figure S3. Refractive index (RI) trace fitting of SEC data to determine SOS content**
- **Table S2. Gaussian fitting parameters used to determine SOS content from the SEC dRI trace**
- **Figure S4. SEC analysis of SOS solutions with varying triblock content**
- **Table S3. Molecular characteristics of SO/SOS samples with varying triblock copolymer content**
- **Figure S5. Water swelling ratio of hydrogels**
- **Figure S6. Model fit to small-angle X-ray scattering (SAXS) data**
- **Table S4. SAXS fitting parameters for varying triblock copolymer content**
- **Figure S7. Stress–strain curves from uniaxial tensile tests of hydrogel fibers**

Characterization of SOS

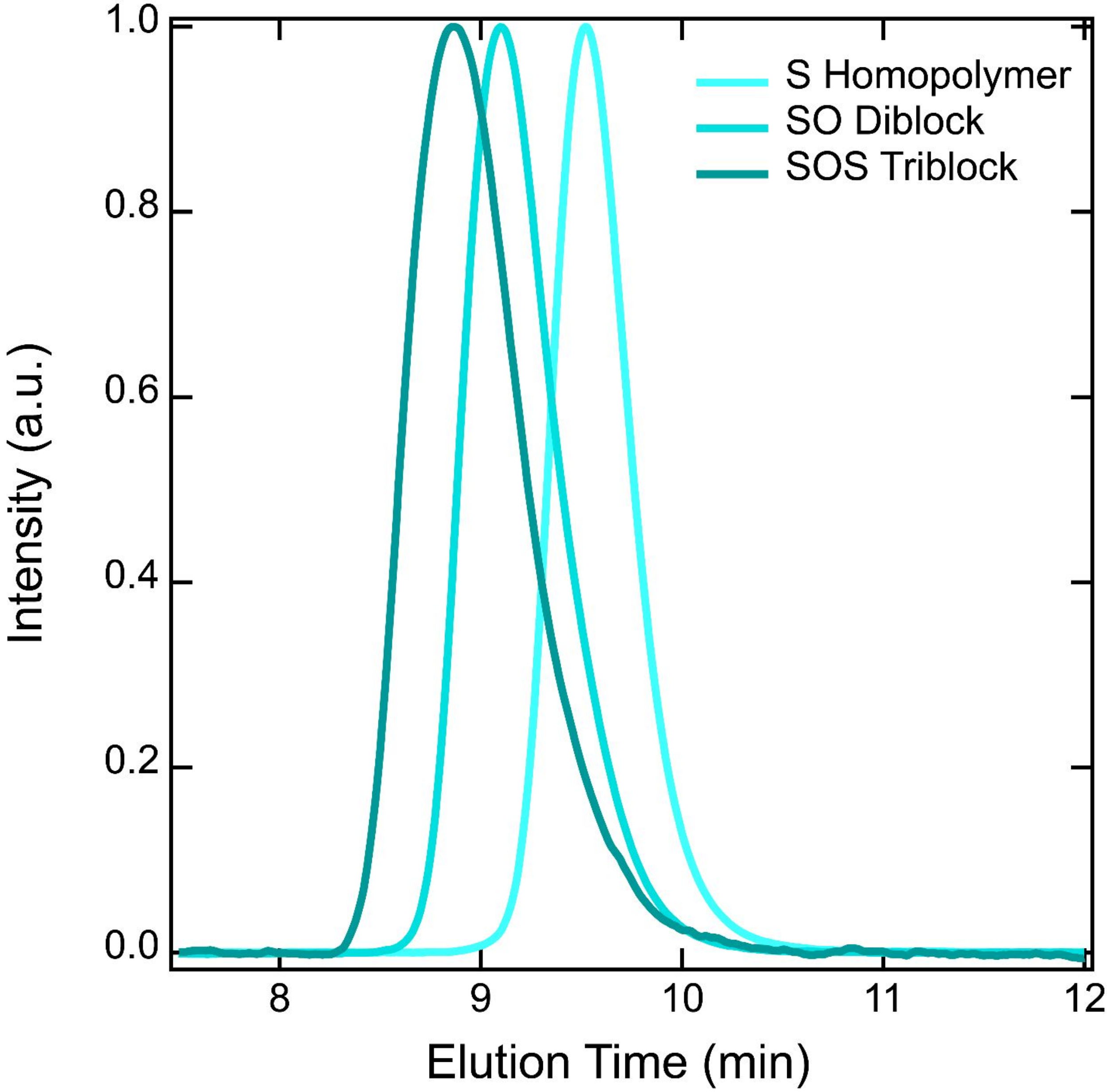

Figure S1. SEC traces for S, SO, and SOS polymers synthesized via living anionic polymerization. SEC measurements were performed using a Tosoh EcoSEC (Tosoh Co.) equipped with a Wyatt Dawn Heleos-II eight angle light scattering detector (Wyatt Technology Corp.) at 40 °C with THF as the mobile phase. The light-scattering detector calibration constant was 3.1620×10^{-5} V^{-1} cm^{-1}.

Table S1. Molecular characteristics of the synthesized polymers

**Polymer**[a]	$M_{n,\,\mathrm{total}}$[b] **(kg/mol)**	$Đ$[c]	f_O[d]	*dn/dc*[e] **(mL/g)**
**S**	41.1	1.01	-	0.185
**SO**	81.4	1.01	0.52	0.124
**SOS**	136.2	1.01	0.52	0.124

[a]S and O denote poly(styrene) (PS) and poly(ethylene oxide) (PEO), respectively; S, SO, and SOS represent the PS homopolymer, PS-PEO diblock copolymer, and PS-PEO-PS triblock copolymer, respectively. [b]Total number-average molecular weight was determined by size-exclusion chromatography (SEC). [c]Dispersity ($Đ = M_w/M_n$) was determined by SEC. [d]The PEO block volume fraction (f_o) was calculated from block compositions determined by ^{1}H NMR using polymer densities reported by Sigma-Aldrich: 1.04 g mL^{-1} for PS and 1.13 g mL^{-1} for PEO at 25 °C. [e]For the PS homopolymer, a previously reported *dn/dc* value in THF was used. For the block copolymers, *dn/dc* values were calculated from the block weight fractions determined by ^{1}H NMR.

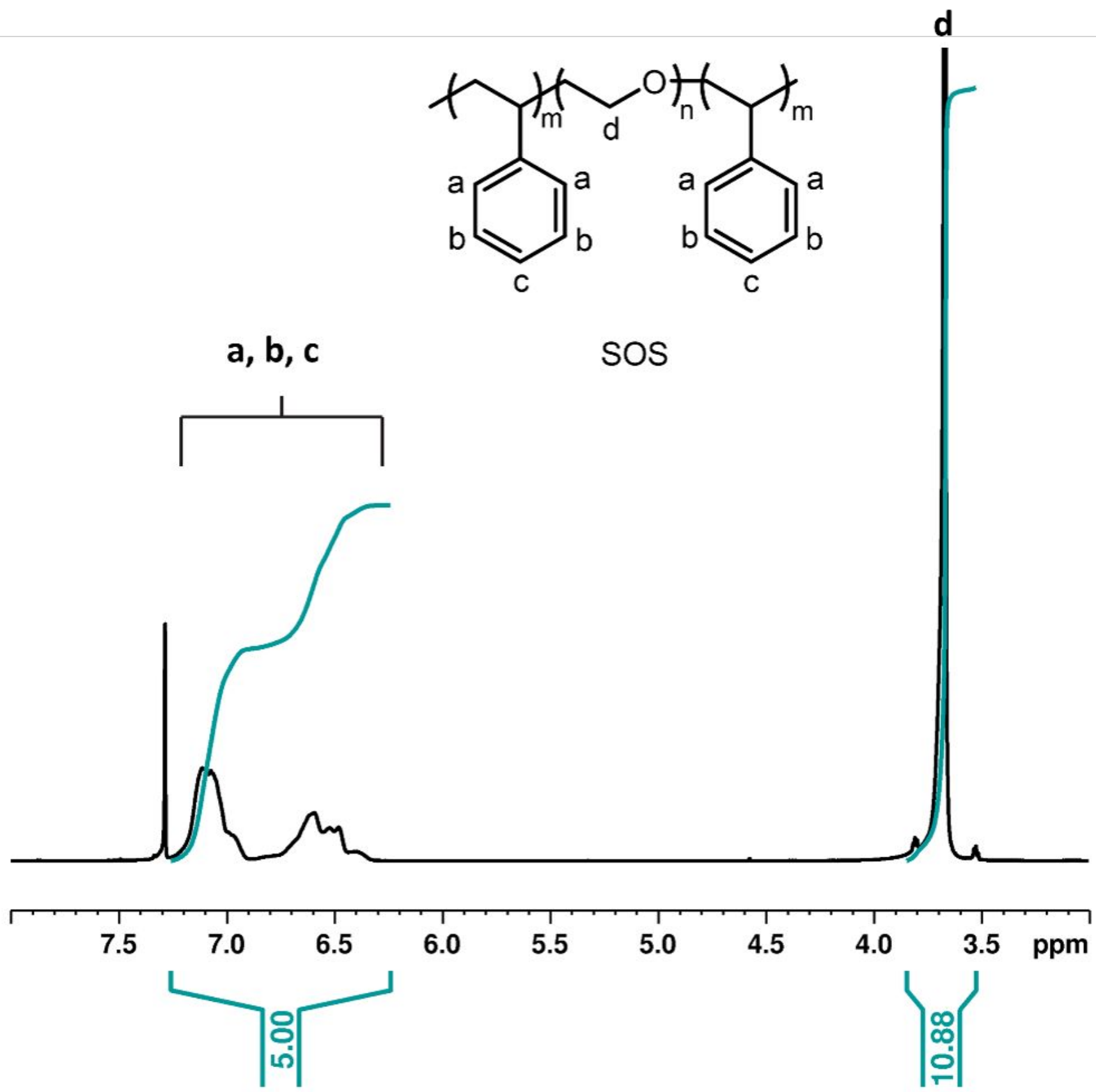

Figure S2. ^{1}H NMR spectrum of SOS.

Composition of SOS from 1H NMR

There are 4 H per ethylene oxide repeat unit ($-CH_2CH_2O-$). Thus, the normalized PEO integration is 10.88 / 4 = 2.72. This corresponds to about 73 mol% PEO.

The integration values are converted to mass:
PS: (5.00/5) × 104.15 = 104.15
PEO: 2.72 × 44.05 = 119.82

Using densities of 1.04 g mL^{-1} for PS and 1.13 g mL^{-1} for PEO, as reported by Sigma-Aldrich:

PS volume = 104.15 / 1.04 = 100.14
PEO volume = 119.82 / 1.13 = 106.03

This gives a PEO volume fraction of:
106.03 / (100.14 + 106.03) = 0.514

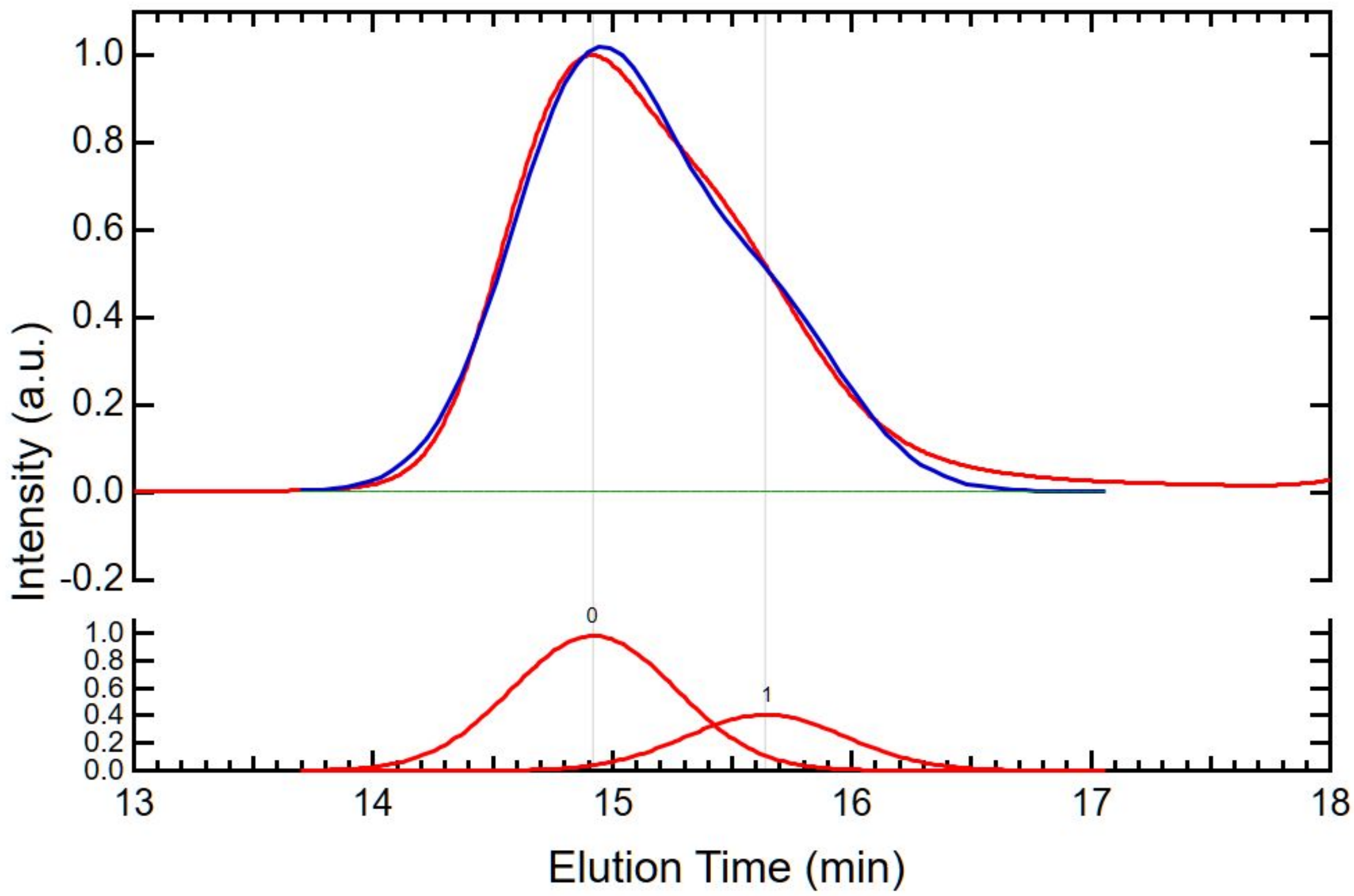

Figure S3. SEC RI trace fit to determine SOS content. Gaussian fit values of the SOS and SO peaks are in **Table S2**.

Table S2. Gaussian fitting parameters used to determine SOS content from the SEC dRI trace

Peak	Assignment	Position (min)	Width (min)	Height (a.u.)	FWHM (min)	Relative Area (%)
0	SOS	14.922	0.4842	0.9777	0.8063	71.2
1	SO	15.642	0.4724	0.4046	0.7866	28.8

Preparation of SOS Hydrogels

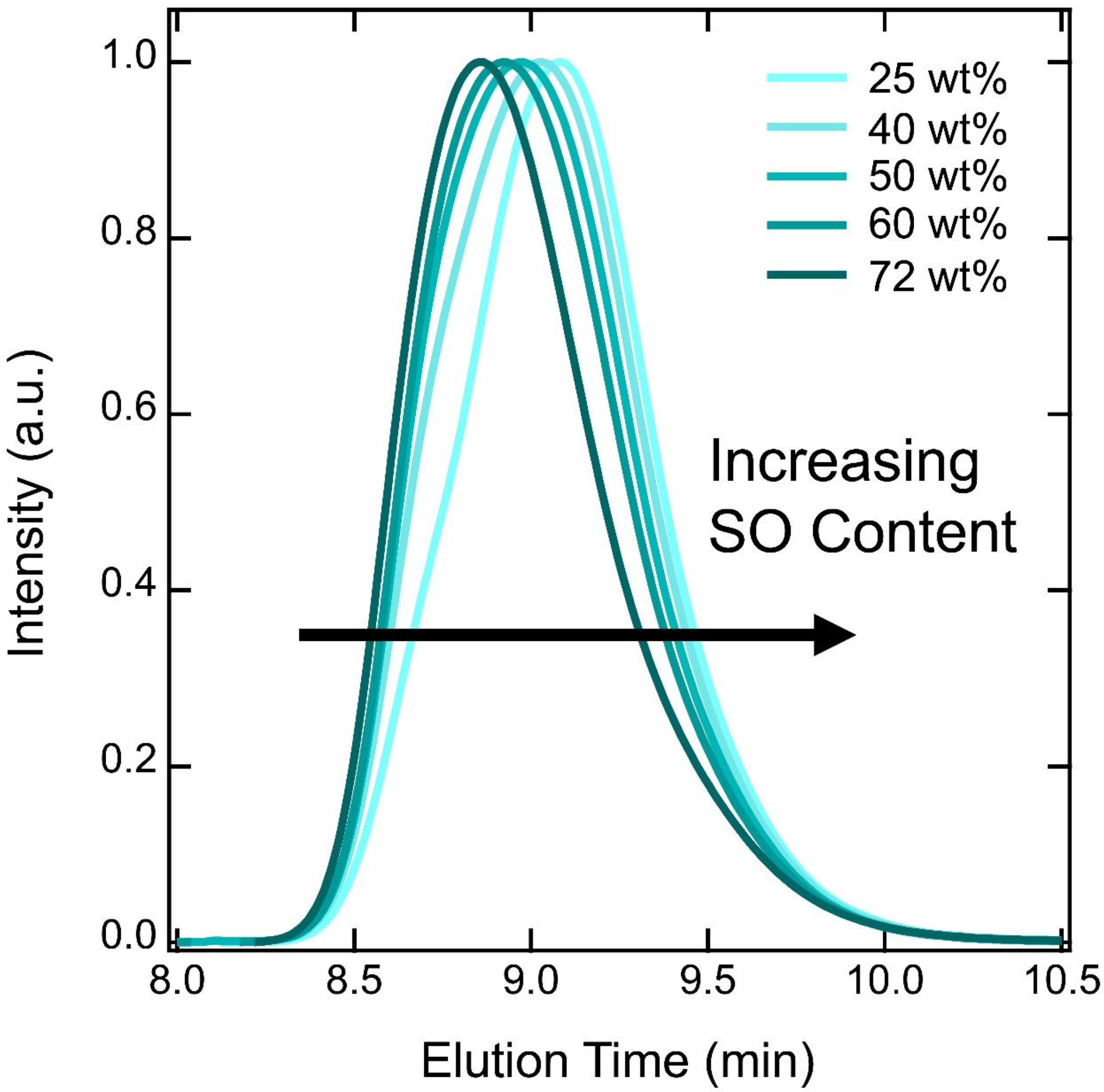

Figure S4. SEC traces of SOS solutions with different amounts of triblock copolymer. There is a change in elution time with decreasing triblock content as expected.

Table S3. Molecular characteristics of SO/SOS samples with varying triblock copolymer content

SOS Content (wt%)	$M_{n,\ total}$ (kg/mol)	*Đ*	*dn/dc* (mL/g)
72	136.2	1.01	0.124
60	115.4	1.05	0.124
50	110.6	1.05	0.124
40	104.5	1.05	0.124
25	95.4	1.03	0.124

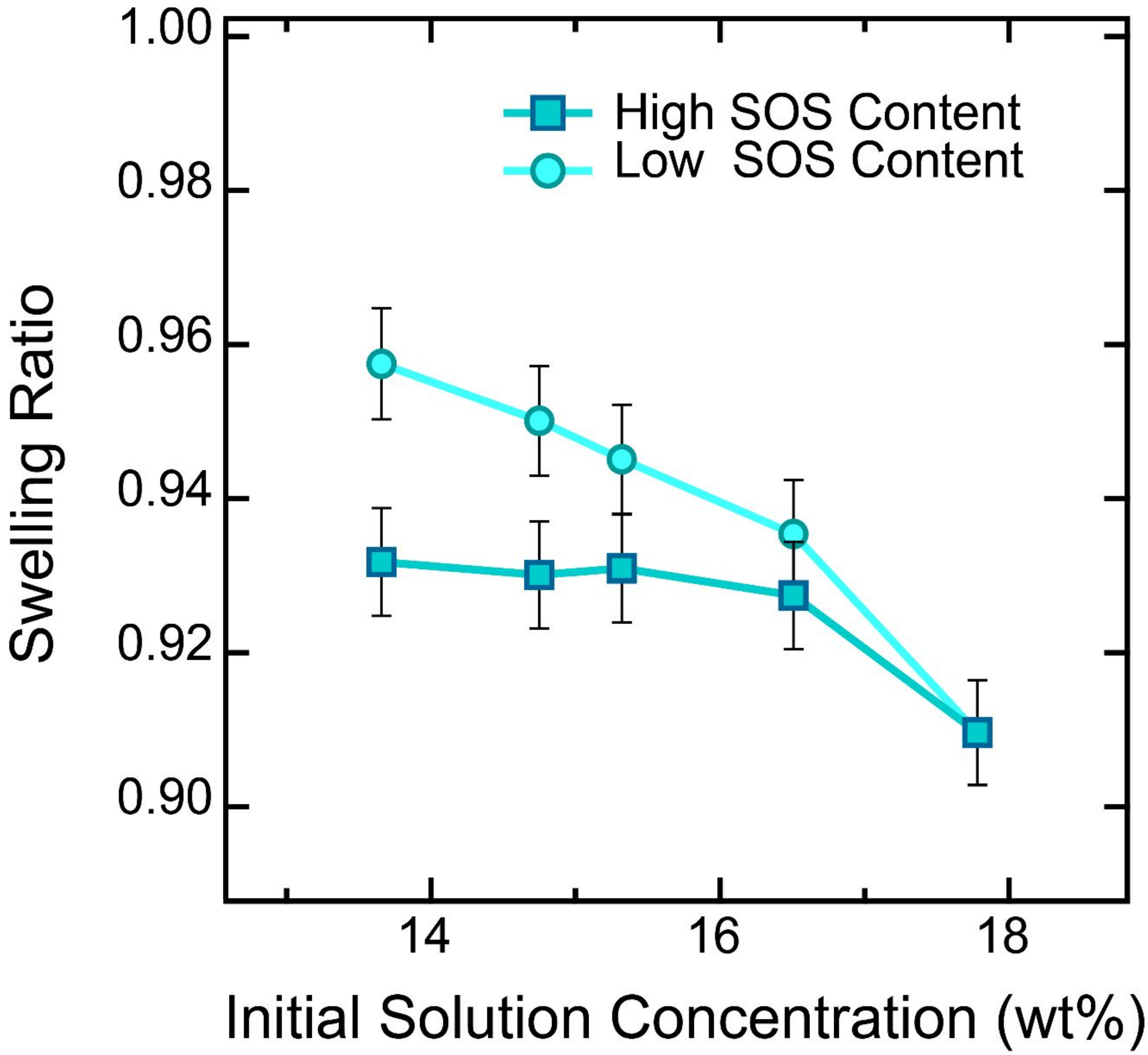

Figure S5. Water swelling ratios for hydrogels prepared using different SOS concentrations for the 72 wt% triblock copolymer sample (high triblock copolymer content) and with varying triblock/diblock copolymer amounts (low triblock content). The results indicate that the SOS concentration alone cannot account for differences in swelling ratio and that increasing the SO content increases water swelling.

SAXS Analysis of the Hydrogels

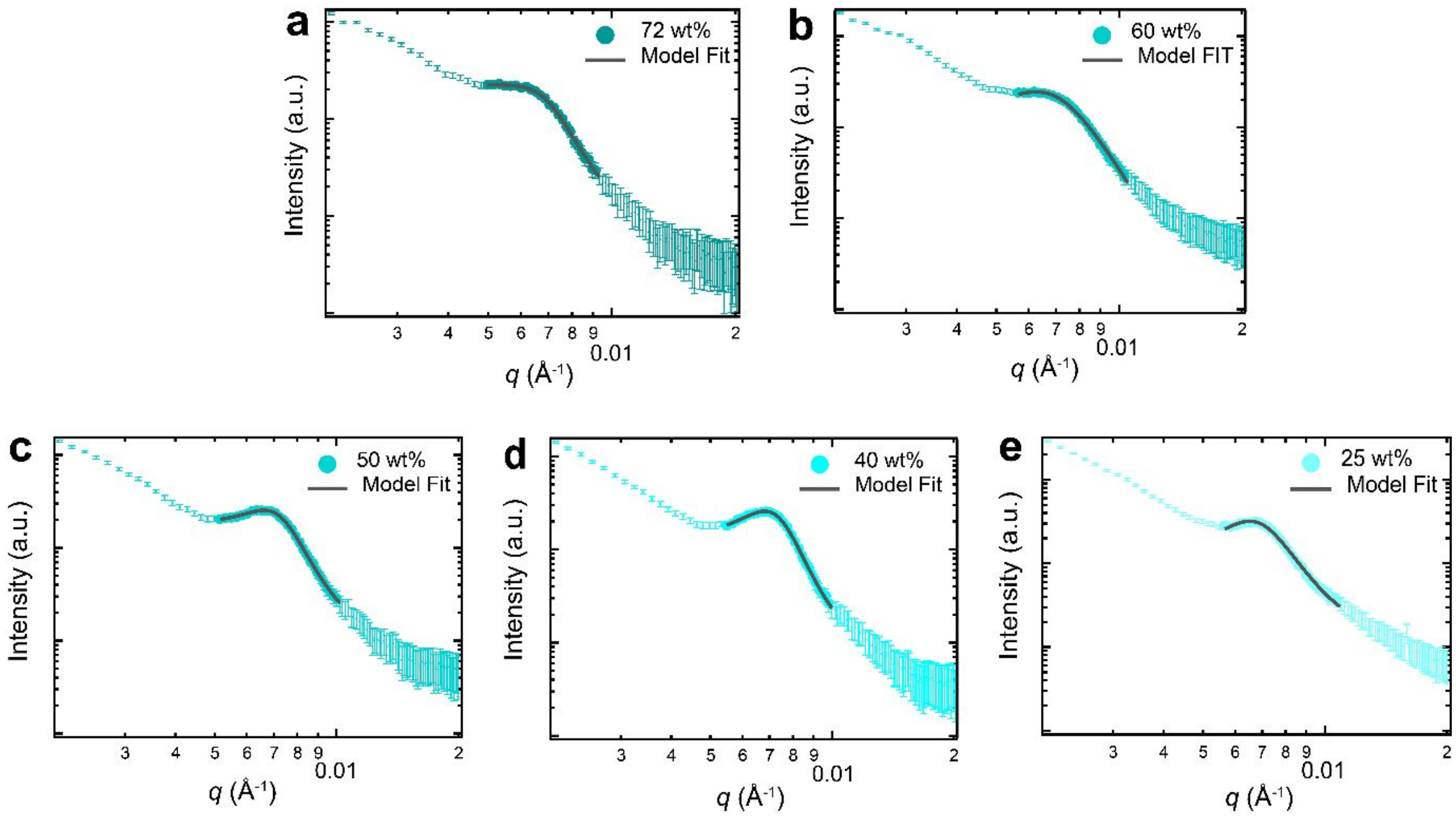

Figure S6. SAXS plots fit with a spheroid form factor and a hard sphere structure factor derived from the Percus-Yevick approximation using Igor Pro equipped with the IRENA package downloaded from Argonne National Lab. Data are shown for (a) 72 wt%, (b) 60 wt%, (c) 50 wt%, (d) 40 wt%, and (e) 25 wt% SOS triblock copolymer. The SAXS fitting parameters are listed in **Table S4**.

Table S4. SAXS fitting parameters for varying triblock copolymer content[a]

SOS Content (wt%)	Mean Micelle Radius (Å)	Micelle Size Standard Deviation (Å)	Hard-Sphere Radius (Å)	Hard-Sphere Volume Fraction
25	297.1 ± 16.2	100.0 ± 74.5	452.0 ± 20.2	0.305 ± 0.021
40	407.9 ± 56.6	116.5 ± 45.0	435.8 ± 12.1	0.352 ± 0.037
50	448.3 ± 121.8	183.2 ± 119.0	440.1 ± 18.9	0.339 ± 0.034
60	324.2 ± 9.2	40.8 ± 11.3	432.9 ± 14.4	0.236 ± 0.027
72	510.9 ± 287.0	159.6 ± 171.6	449.2 ± 78.4	0.300 ± 0.123

[a]SAXS data were fitted using the IRENA package in Igor Pro with a spheroid form factor and a hard-sphere structure factor derived from the Percus–Yevick approximation.

Uniaxial Extension of the Hydrogels

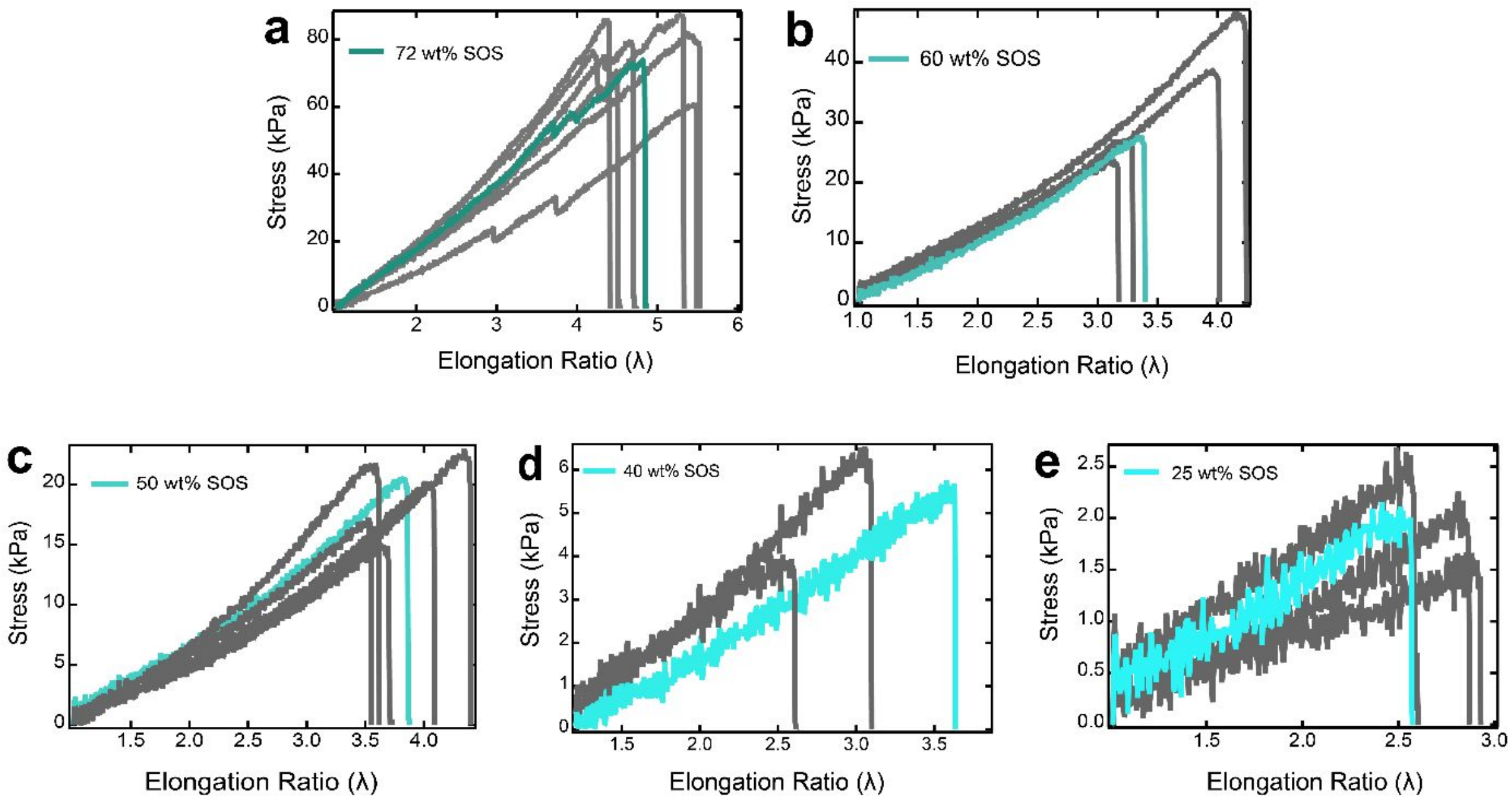

Figure S7. Stress–strain curves from uniaxial tensile tests of hydrogel fibers with a) 72 wt%, b) 60 wt%, c) 50 wt%, d) 40 wt%, and e) 25 wt% SOS. Stress-strain curves shown in teal are the curves shown in **Figure 5** of the main text.
